# The Cytoplasmic Tail of FPC Antagonizes the Full-Length Protein in the Regulation of mTOR Pathway

**DOI:** 10.1371/journal.pone.0095630

**Published:** 2014-05-22

**Authors:** Shixuan Wang, Maoqing Wu, Gang Yao, Jingjing Zhang, Jing Zhou

**Affiliations:** Renal Division, Department of Medicine and Center of Polycystic Kidney Disease, Brigham and Women's Hospital and Harvard Medical School, Boston, Massachusetts, United States of America; University of Geneva, Switzerland

## Abstract

FPC (fibrocystin or polyductin) is a single transmembrane receptor-like protein, responsible for the human autosomal recessive polycystic kidney disease (ARPKD). It was recently proposed that FPC undergoes a Notch-like cleavage and subsequently the cleaved carboxy(C)-terminal fragment translocates to the nucleus. To study the functions of the isolated C-tail, we expressed the intracellular domain of human FPC (hICD) in renal epithelial cells. By 3-dimensional (3D) tubulogenesis assay, we found that in contrast to tubule-like structures formed from control cells, hICD-expressing cells exclusively formed cyst-like structures. By western blotting, we showed that the Akt/mTOR pathway, indicated by increased phosphorylation of Akt at serine 473 and S6 kinase 1 at threonine 389, was constitutively activated in hICD-expressing cells, similar to that in FPC knockdown cells and ARPKD kidneys. Moreover, application of mTOR inhibitor rapamycin reduced the size of the cyst-like structures formed by hICD-expressing cells. Application of either LY294002 or wortmannin inhibited the activation of both S6K1 and Akt. Expression of full-length FPC inhibited the activation of S6 and S6 kinase whereas co-expression of hICD with full-length FPC antagonized the inhibitory effect of full-length FPC on mTOR. Taken together, we propose that FPC modulates the PI3K/Akt/mTOR pathway and the cleaved C-tail regulates the function of the full-length protein.

## Introduction

The most common forms of polycystic kidney disease (PKD) in humans are autosomal dominant and recessive PKD (ADPKD and ARPKD). ADPKD is the adult form of the disease, caused by mutations in either *PKD1* or *PKD2*, the genes respectively encoding polycystin-1 and polycystin-2 which form a receptor-channel complex [1], [2]. ARPKD is the pediatric form resulting from mutations in *PKHD1*, the gene encoding fibrocystin/polyductin (FPC) [3]–[5]. FPC is a single transmembrane protein (4,074 amino acids, aa) with a large extracellular domain (3,859 aa), a single transmembrane segment (23 aa), and a small intracellular C-tail (hICD, 192 aa). Abnormalities in cell proliferation, apoptosis, and extracellular matrix (ECM) are among the features of PKD [1], [2]. FPC interacts with polycystin-2, both of which are localized to the primary cilia of kidney epithelial cells [6]–[12] where they participate cellular mechanosensation [11], [13].

The C-terminus of FPC has been suggested to harbor an atypical 25-residue nuclear localization signal sequence and the cleaved fragment translocates into the nuclei in mIMCD-3 and MDCK cells [14], [15]. An 18-residue sequence at the C-terminus of FPC was recently identified to be responsible for its ciliary targeting by transient expression of a series of deletion constructs in mIMCD-3 cells [16]. However, the functions of the full-length FPC and its cleaved C-tail remain unknown. In this study, we report that hICD expression leads to activation of the phosphoinositide 3-kinase (PI3K)-Akt-mammalian target of rapamycin (mTOR) pathway and formation of cyst-like structures in 3D collagen cultures. Furthermore, we show that mTOR inhibitor rapamycin reduces the sizes of the cyst-like structures formed by hICD-expressing cells. Our data suggest that FPC modulates the activities of the PI3K/Akt/mTOR pathway and that the expression of FPC C-tail may antagonize the function of the full-length protein and promote cyst formation.

## Results

### Expression of hICD

**Figure 1:**
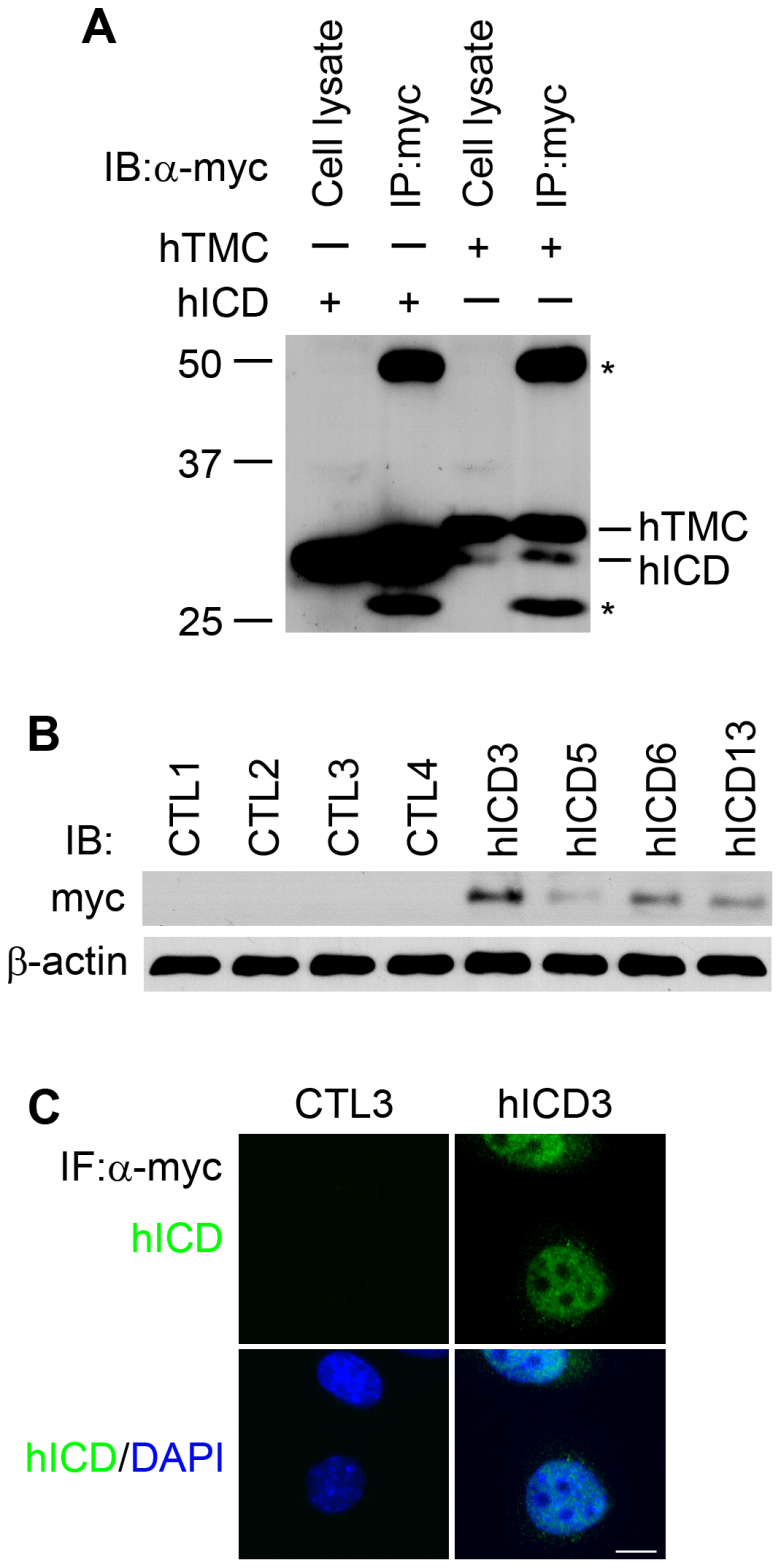
Establishment of hICD-expressing stable cells. (A) Transient expression of hTMC construct in HEK293 cells produced, in addition to the expected TMC fragment, a band similar in size to the intracellular domain of FPC. * Ig heavy and light chains. (B) Western blotting showing the levels of hICD in four mIMCD-3 cell lines stably expressing hICD (hICD3, 5, 6, 13), along with four empty vector control cell lines (CTL1, 2, 3, 4). IB, immunoblotting. (C) FPC C-tail was mainly localized in the nuclei of mIMCD-3 cells (hICD3) compared to the control cell line (CTL3). IF, immunofluorescence. Scale bar, 5 µm.

We transiently expressed either hICD alone or the intracellular domain with its preceding transmembrane segment (hTMC) in HEK293 cells (Figure 1, Figure S1). Two close bands at ∼30 kDa were detected in hTMC-transfected cells. In addition to the expected upper higher molecular weight one, the lower molecular weight band had an identical migration pattern to that in hICD-transfected cells (Figure 1A). This result is consistent with the finding that hICD can be cleaved from full-length FPC [14], [15] and the size of the cleaved C-tail is similar to that of hICD. In order to study the function of the cleaved fragment, we established four mIMCD-3 cell lines stably expressing hICD (Figure 1B, C). In line with the previous reports [14], [15], hICD was mainly localized in the nuclei of mIMCD-3 cells with some signals in the cytoplasm.

### Expression of hICD leads to cystogenesis

**Figure 2:**
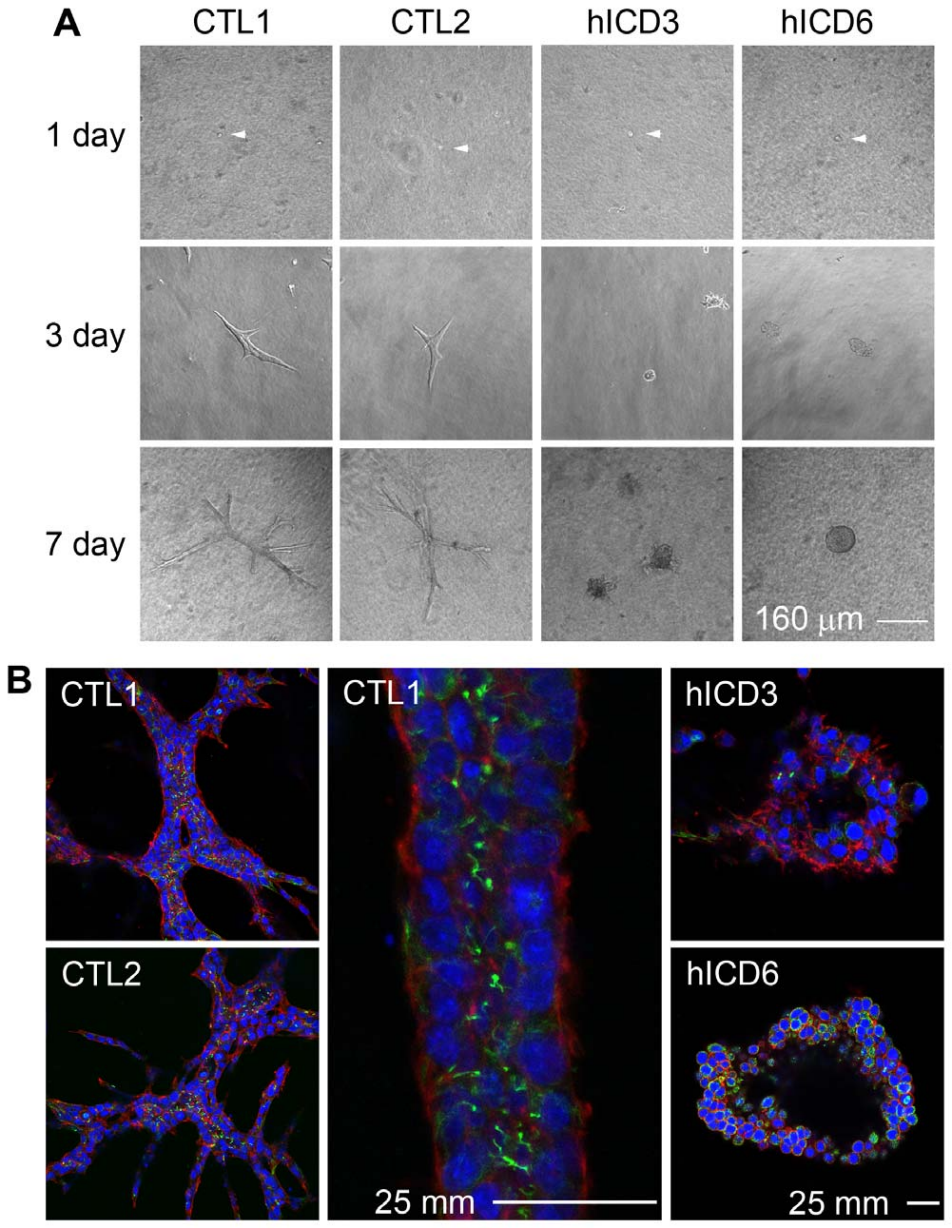
FPC C-tail expression caused cystogenesis in 3D culture. hICD cells from all four cell lines (3, 5, 6, 13), in parallel with cells from four control lines, were cultured in collagen I gels. All hICD-expressing lines (3, 6, 13, 100%; 5, <100%) formed cyst-like structures in contrast to tubule-like structures formed by control cells. The representative pictures were presented (A, B). (A) Tubulogenesis of both control (CTL1, CTL2) and hICD (hICD3, hICD6) cells at 1, 3 and 7 days after seeding in collagen I gels. At day 3 and 7, in contrast to control cells, which developed tubule-like structures (100%), hICD-expressing cells formed cyst-like structures (100%). (B) Confocal microscopy reveals the presence of a lumen in the tubule-like structure developed from control cells (CTL1, 2) and a central cavity in cyst-like structures in hICD-expressing cells (CTL3, 6) after 8-day culture. A segment of a tubule was shown with a higher magnification. HGF was not used in this experiment. Red, rhodamine phalloidin; green, acetylated α-tubulin; blue, DAPI.

Tubulogenesis assay in collagen I gel or Matrigel is commonly used to mimic the *in vivo* morphogenesis of kidney tubules. To explore the role of hICD in tubulogenesis, we cultured hICD-expressing cells in 3D collagen gels for up to 8 days. Remarkably, all four mIMCD-3 cell lines stably expressing hICD formed cyst-like structures (hICD3, 6, 13, 100%; hICD5, <100%), in striking contrast to all four empty vector-transfected control cell lines (CTL1, 2, 3, 4, 100%) which formed tubule-like structures irrespective of the absence or presence of hepatic growth factor (HGF) (Figure 2A). To further analyze the cyst-like structures formed by hICD-expressing cells, we performed in-gel staining of these structures with antibodies to acetylated α-tubulin and phalloidin. Confocal microscopy revealed that all cyst-like structures possessed a central cavity (Figure 2B). All control cells developed tubule-like structures with primary cilia protruding towards the lumen (Figure 2B).

### Constitutive mTOR activation in hICD-expressing cells

**Figure 3:**
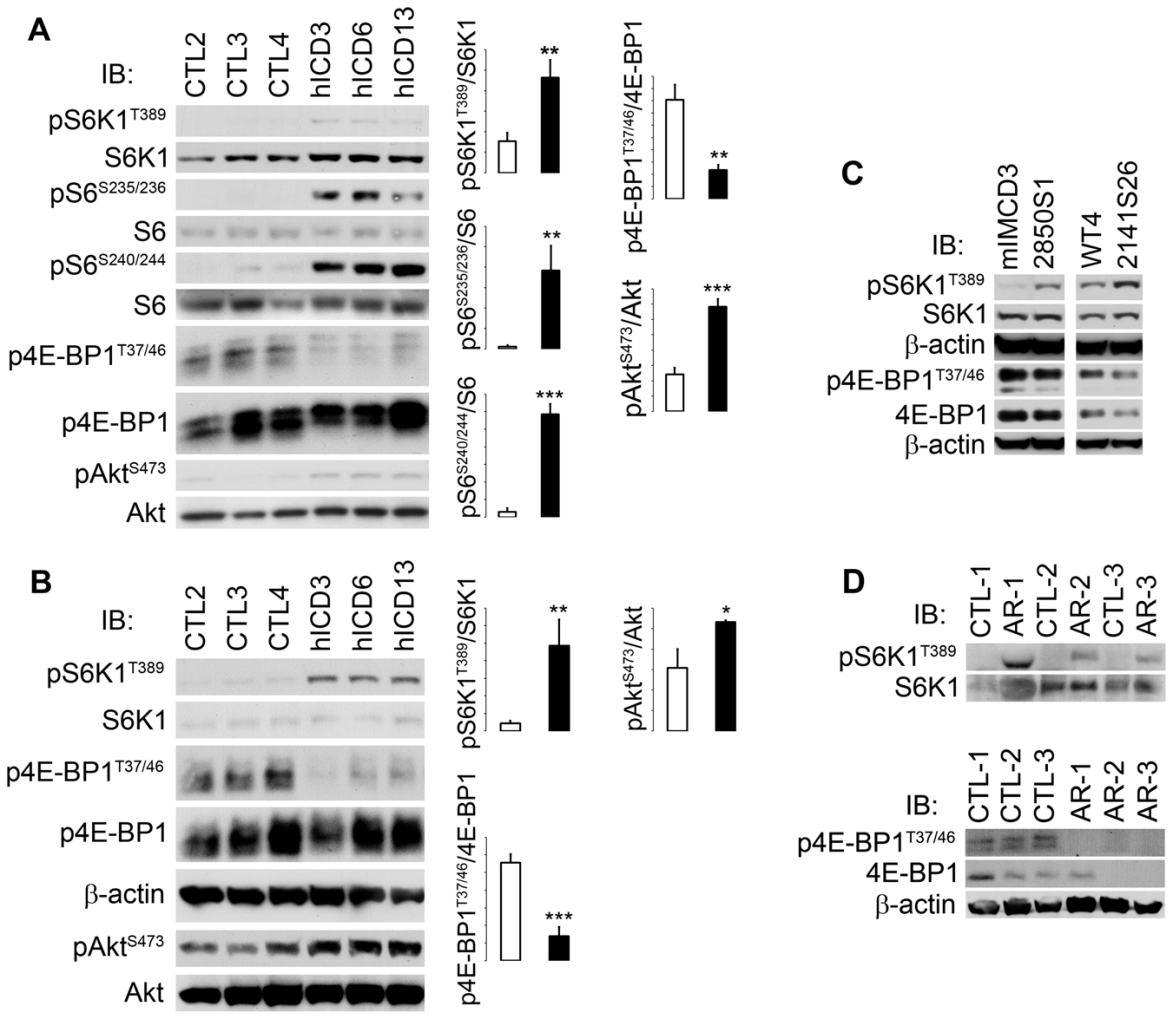
mTOR activation in hICD-expressing cells and ARPKD kidneys. (A) In contrast to that in control cells, pS6K1^T389^, pS6^S235/236^, pS6^S240/244^, and pAkt^S473^ were activated and p4E-BP1^T37/46^ was suppressed in serum-starved hICD-expressing cells (DMEM/F12/0.5% serum for 16 h). (B) In amino acid-starved hICD-expressing cells, Akt^S473^ and S6K1^T389^ were hyperphosphorylated compared to those in controls, whereas 4E-BP1^T37/46^ was de-phosphorylated. The bar graphs represented the activation of specified proteins expressed as a ratio of the phosphorylation form/total protein (N = 3). (C) In two pairs of FPC knockdown cells (2850S1 and 2141S26), S6K1^T389^ was hyperphosphorylated under serum-starved conditions. The numbers on the Y-axis were arbitrary units of the bands intensity ratio. (D) S6K1^T389^ was highly phosphorylated while phosphorylation of 4E-BP1^T37/46^ was reduced in ARPKD kidneys (AR-1, 2, 3), compared to normal controls (CTL-1, -2, -3). Open bars, control cells; filled bars, hICD-expressing cells. Values were expressed as mean ± SD. * p<0.05, ** p<0.01, *** p<0.001.

In order to study the mechanism of cystogenesis, we examined mTOR signaling pathway in hICD-expressing cells. Aberrant activation of S6 kinase 1 (S6K1), as indicated by phosphorylation at threonine 389 (S6K1^T389^), was observed in hICD-expressing cells, compared to that in control cells under serum starvation conditions (Figure 3A). Consistently, S6, the substrate of S6K1, was phosphorylated at serines 235/236 and 240/244 (S6^S235/236, S240/244^). Because both growth factors and amino acids independently regulate mTOR in a distinct manner and amino acids regulate mTOR probably through the cytoplasmic Rag GTPase [17], [18], we went on to test the effects of amino acids on mTOR signal cascade. Compared to that in control cells, amino acids removal failed to down-regulate mTOR activation in hICD-expressing cells (Figure 3B).

### Activation of mTOR signaling in FPC knockdown cells and ARPKD kidneys

Constitutive S6K1^T389^ activation was also found in FPC knockdown mIMCD-3 cells [13] (Figure 3C) and in kidney tissues from 3 ARPKD patients (Figure 3D). Because Akt is a key regulator of mTOR activity, we studied phosphorylation of Akt^S473^ and found that it was constitutively hyperphosphorylated in hICD-expressing cells (Figure 3A, B). Interestingly we observed a decrease in 4E-BP1^T37/46^ phosphorylation in hICD-expressing cells compared to control cells (Figure 3A, B). This decrease was also detected in FPC knockdown cells and in kidney tissues from patients with ARPKD (Figure 3C, D).

### PI3K is responsible for Akt/mTOR activation

**Figure 4:**
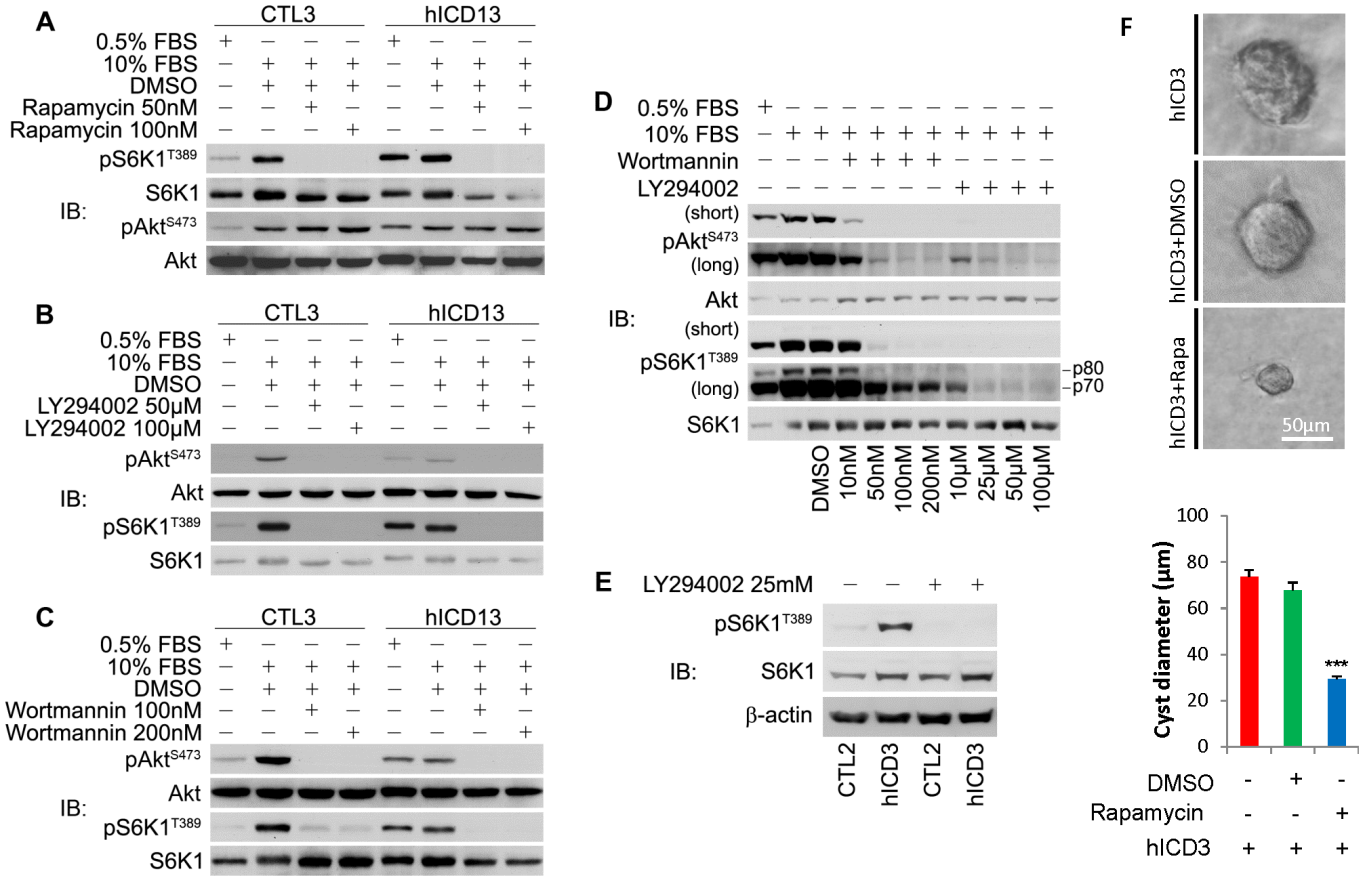
mTOR activation in hICD-expressing cells can be blocked by PI3K inhibitors and rapamycin. (A) Application of rapamycin completely blocked S6K1^T389^ phosphorylation in both hICD-expressing and control cells, while the phosphorylation of Akt^S473^ was not affected as expected. (B, C) PI3K inhibitors LY294002 and Wortmannin displayed inhibitory effects on both pAkt^S473^ and pS6K1^T389^. (D) The activation of both pAkt^S473^ and pS6K1^T389^ were sensitive to a variety of concentrations of wortmannin and LY294002. Short- and long-time exposure of western blotting was displayed. (E) In the absence of serum, pS6K1^T389^ was de-phosphorylated with LY294002 treatment in both control and hICD-expressing cells. (F) Effects of mTOR inhibitor rapamycin treatment in 3D collagen gels. Cysts were smaller after 11-day treatment of rapamycin (10 nM), in contrast to vehicle (DMSO)-treated or non-treated ones. Scale bar, 50 µm. Values were expressed as mean ± SE. *** p<0.001.

To investigate the mechanisms leading to the activation of mTOR and Akt in hICD-expressing cells, we examined the activity of PI3K, the major upstream regulator of Akt, using inhibitors of PI3K (wortmannin and LY294002). As reported [19], rapamycin completely inhibited S6K1 activity but not Akt phosphorylation (Figure 4A). Application of either LY294002 or wortmannin inhibited the activation of both S6K1 and Akt (Figure 4B, C). Because there was little information on the dosage of these inhibitors in mIMCD-3 cells, we examined the effects of different doses of PI3K inhibitors. We found that S6K1 activity in hICD-expressing cells was highly sensitive to these inhibitors and the inhibition of pAkt^S473^ was correlated with the decrement of pS6K^T389^ (Figure 4D). The inhibitory effects of LY294002 on S6K1 in hICD-expressing cells remained in the absence of serum (Figure 4E).

### Treatment of mTOR inhibitor rapamycin inhibits cyst growth

In order to determine whether sustained activation of mTOR signaling was responsible for abnormal branching morphogenesis leading to cystogenesis, we applied the mTOR inhibitor rapamycin to hICD-expressing cells cultured in 3D collagen gels. We found that cyst-like structures became strikingly smaller in treated hICD-expressing cells than those in non-treated or DMSO-treated controls (Figure 4F).

### Cell proliferation, cell cycle progression, and apoptosis in hICD-expressing cells

**Figure 5:**
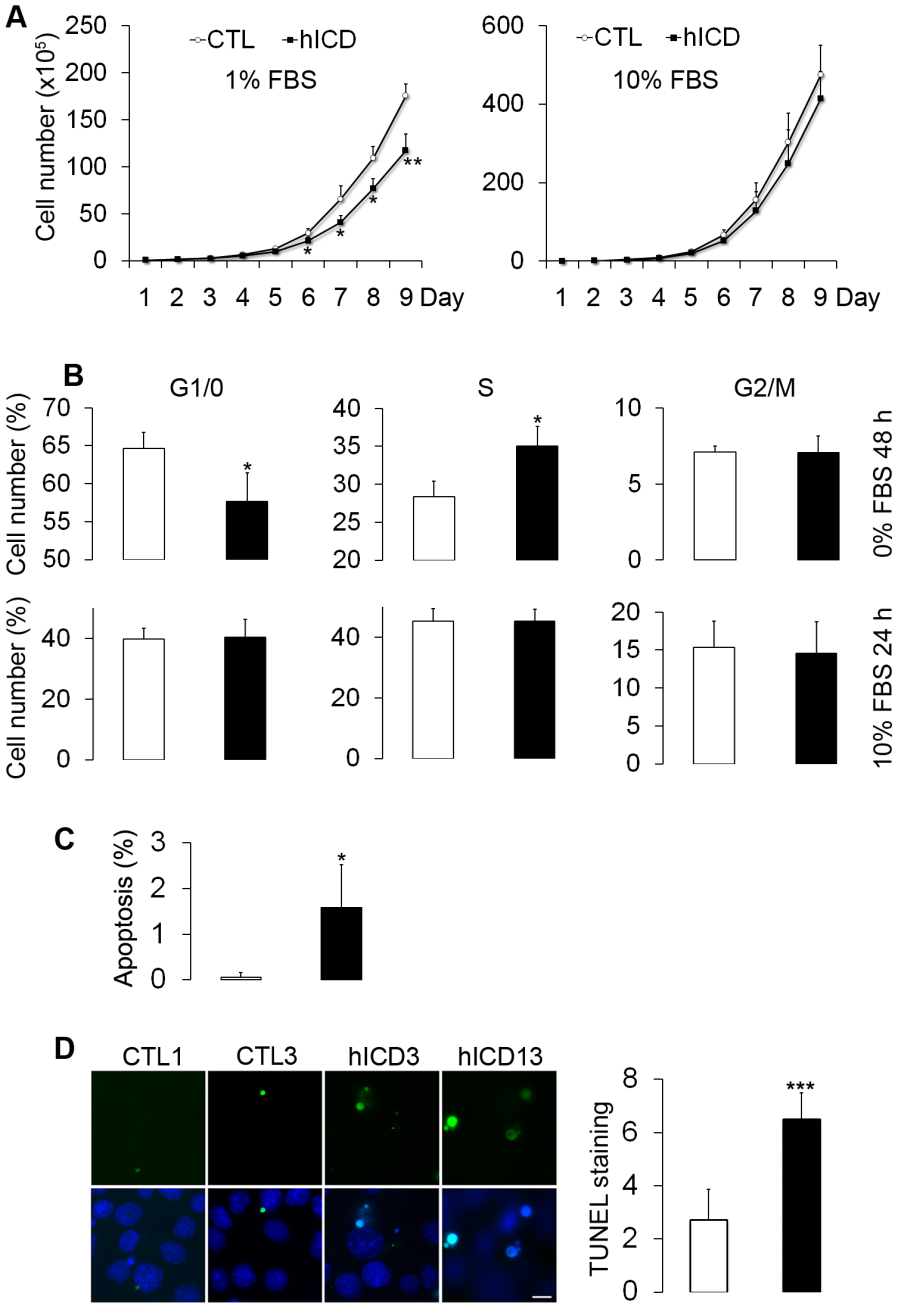
Decreased cell proliferation rate and increased apoptosis in hICD-expressing cells. (A) The number of hICD-expressing cells was significantly lower than that of control cells when cultured in 1% but not in 10% serum-containing medium (N = 3 hICD3, 6, 13). One representative of three experiments was presented. (B) With flow cytometry analysis, more hICD-expressing cells passed the restriction point in G1/0-phase but were arrested in S-phase after serum removal for 48 h. Addition of 10% serum for 24 h completely compromised the effect of hICD expression on the cell cycle profile (N = 3 hICD3, 6, 13). One representative of three experiments was presented. (C) More apoptotic hICD-expressing cells were found in the absence of serum by flow cytometry analysis (N = 3 hICD3, 6, 13). One representative of at least three experiments was presented. (D) Three control and three hICD cell lines were used for this experiment. Pictures from two control and hICD cell lines were presented. TUNEL staining assay confirmed an increase of apoptotic hICD-expressing cells. Bar graph represented statistical significance (N = 200 cells from three controls and hICD cell lines). Open bars, control cells; filled bars, hICD-expressing cells. Values were expressed as mean ± SD. * p<0.05, ** p<0.01, *** p<0.001. Scale bar, 10 µm.

Increased cell proliferation and apoptosis have been regarded as the pivotal mechanisms of PKD. We therefore examined cell growth and apoptosis in hICD-expressing cells. The doubling time of hICD-expressing cells was a little longer than that of controls, especially when cultured in 1% serum-containing medium (Figure 5A). We further analyzed the cell cycle profile of these cells by flow cytometry. After serum withdrawal for 2 days, a large proportion of hICD-expressing cells failed to exit the cell cycle and remained in the S-phase, in contrast to the control cells. After replenishment of 10% serum, the difference between hICD-expressing and control cells became negligible (Figure 5B). In the absence of serum, there was an increase of apoptosis in hICD-expressing cells by flow cytometry (Figure 5C). To confirm these data, we performed TUNEL assay in the presence and absence of 10% serum. Compared to control cells, there was a significant increase in apoptosis in hICD-expressing cells especially in serum-free conditions (Figure 5D).

### The cytoplasmic C-tail of FPC antagonizes the inhibitory effect of full-length FPC on mTOR

**Figure 6:**
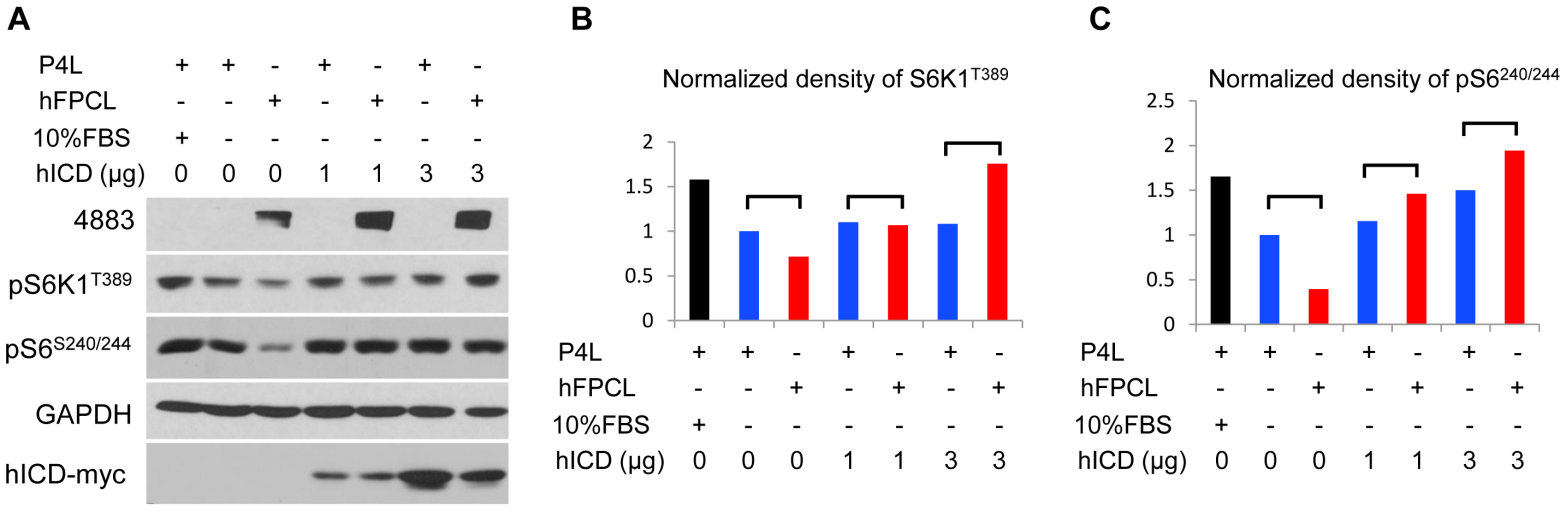
Opposite roles of full-length FPC and C-tail on mTOR. (A) Human full-length FPC- (hFPCL)- and control empty vector (P4L)-expressing LLC-PK1 cells were transfected with various amounts of hICD plasmid DNA (0, 1, 3 µg). Twenty-four hours after transfection, cells were serum starved for 24 h before protein extraction. Expression of full-length FPC inhibited the phosphorylation of p70S6K^T389^ and pS6^S240/244^ in comparison to the controls, while hICD expression antagonized the inhibition of full-length FPC on p70S6K^T389^ and pS6^S240/244^ in a dose-dependent fashion. One representative experiment was shown.

To understand the mechanistic action of hICD, we transiently expressed hICD in a kidney proximal tubule cell line (LLC-PK1) stably expressing full-length FPC (hFPCL) [12]. We found that expression of full-length FPC inhibited mTOR signaling exemplified by reduced activation of S6K1^T389^ (Figure 6A, B). A stronger inhibitory effect of full-length FPC on mTOR was seen on S6^S240/244^ activation (Figure 6A, C). Coexpression of hICD with full-length FPC, however, relieved the inhibitory effects of full-length FPC on mTOR activation, or even promoted mTOR activation (at 3 µg) (Figure 6).

## Discussion

In this study, we report for the first time that FPC is capable of modulating the PI3K/Akt/mTOR pathway. Expression of full-length FPC inhibited the activation of mTOR. Yet overexpression of FPC C-tail antagonized the function of full-length protein, leading to the activation of PI3K, Akt and mTOR pathways, defects in cell cycle progression and cell survival, and cystogenesis. Activation of the mTOR pathway was confirmed in FPC knockdown cells and kidneys from human ARPKD patients. Pharmacological inhibition of PI3K and mTOR partially inhibited cystogenesis, confirming the contribution of PI3K/Akt/mTOR activation to cystogenesis of hICD-expressing cells.

The cleavage of FPC C-tail has been observed when full-length FPC is overexpressed [14], [15]. We found here that hTMC, the last 225-residue of human FPC which contains 33 amino acids of the transmembrane domain and 192 residues of the cytoplasmic tail, was further cleaved and produced a fragment that was similar in size to the intracellular tail cleaved from the full-length FPC [14], [15]. These data suggest that the large extracellular portion of FPC is not required for cleavage to occur. Because both the full-length FPC and the hTMC constructs were expressed in non-nuclear compartments of the cell, the cleavage of the FPC C-tail likely occurred in the cytoplasm or on the cell membrane by an unidentified enzyme(s). FPC is detected on the primary cilia with antibodies to both N- and C-termini of FPC [9], [12], we thus believe that FPC undergoes a C-tail cleavage under defined conditions and some of the cleaved fragments translocate into the nucleus [14], [15]. Notably, our human free FPC C-tail was not found on the primary cilia, in contrast to the mouse FPC C-tail constructs used for the study of ciliary targeting sequence which contains additional nine residues (LSCLVCCWF) in the transmembrane segment of FPC [16], although nuclear FPC C-tail signals were also observed in that study [16].

Sustained phosphorylation of S6K1 and S6 in hICD-expressing cells implied the activation of mTOR signaling pathway. The mTOR signaling pathway has, in the past several years, gained much attention in the PKD research field. It was reported that mTOR is activated in cyst-lining cells in human ADPKD kidneys and inhibition of mTOR pathway reverses renal cystogenesis in animal models [20], although clinical trials with rapamycin in human ADPKD patients were disappointing [21]. The mTOR protein binds to other proteins to form a rapamycin-sensitive complex (mTORC1) or a rapamycin-insensitive complex (mTORC2) [19], [22]. The mTORC1 is regulated by upstream factors such as nutrients, growth factors, and energy metabolism, and can phosphorylate a series of downstream substrates. Upstream regulators of mTOR include Akt (PKB), PI3K/PTEN, TSC1/2, AMPK, and Rheb [23], [24]. The mTORC2 has been suggested to mainly regulate the actin cytoskeleton through Rho family of small GTPases and protein kinase C [25]. In our experiments, we found that mTOR activation was associated with constitutive phosphorylation of Akt^S473^. As Akt^S473^ is phosphorylated by mTORC2 [26]–[28], we assumed that Akt^S473^ phosphorylated by mTORC2 further activated mTORC1. Together with the data from the application of PI3K inhibitors, these data suggest that hICD may activate mTORC1 through PI3K-Akt or mTORC2-Akt pathways.

We found that the full-length FPC inhibited mTOR activity, which was consistent with our finding on the aberrant mTOR activation in FPC knockdown cells and kidney samples from ARPKD patients. Overexpression of free FPC C-tail antagonized the full-length protein function and led to mTOR activation. The mechanisms by which full-length FPC and free C-tail regulated mTOR activity may be different, as the C-tail was largely located in the nucleus while the full-length was mostly membrane-associated. Overexpression of FPC C-tail may disrupt the full-length FPC mediated signaling pathways by competitively occupying the binding sites of its interacting proteins, or sequestering respective proteins into the nucleus and consequently affecting the physiological function of full-length FPC. When expressed at a high level, FPC-C-tail overexpression may activate mTOR through an additional mechanism(s). We cannot exclude the possibility that hICD activated the mTOR signaling pathway through a gain-of-function mechanism in the cell nucleus. Because the level of endogenously cleaved FPC C-tail is, like that of Notch, too low to be detectible [14], we propose that under normal physiologically conditions, full-length FPC inhibits mTOR signaling, whereas C-tail cleavage is facilitated under specific conditions.

FPC C-tail-expressing cells exclusively formed cyst-like structures, suggesting that the consequence of hICD expression is similar to that of FPC knockdown [29]. Application of mTOR inhibitor rapamycin reduced the size of cyst-like structures formed by hICD expressing cells, which indicates that the mTOR signaling pathway is a potential therapeutic target for ARPKD.

It is generally believed that cyst-lining cells are more proliferative based on markers such as proliferative cell nuclear antigen. Indeed, under low serum conditions, we found that, compared to control cells, there were more C-tail-expressing cells situated in the S phase of the cell cycle, and fewer cells in the G0/G1 phase, indicating that FPC regulates G1-S progression. We have previously shown that FPC regulates centrosome duplication and mitotic spindle assembly during cell division [12]. Here we found that the expression of hICD was associated with an increase in apoptosis, a feature of ARPKD [30] and FPC knockdown cells [29]. Taken together, the increased apoptosis may be a consequence of defects in the cell cycle and constitutive activation of mTOR, as mTOR pathway can either inhibit or promote apoptosis depending on the cell context [31].

In summary, we show here that ARPKD protein FPC regulated PI3K/Akt/mTOR signaling pathway. Further research is needed to understand how FPC and other PKD proteins regulate this signaling pathway in a coordinated manner.

## Materials and Methods

### Antibodies

Antibodies to a number of proteins in the mTOR pathway (pS6K1^T389^, S6K1, pS6^S235/236^, pS6^S240/244^, S6, p4E-BP1^T37/46^, 4E-BP1, pAkt^S473^, Akt) were obtained from Cell Signaling Technologies (Cambridge, MA, USA). β-actin and acetylated α-tubulin antibodies were bought from Sigma (St. Louis, MO, USA). Antibodies to c-myc (9E10) were purchased from the BWH-BRI Core Facility. Rhodamine phalloidin was from Invitrogen (Carlsbad, CA, USA).

### DNA constructs, stable cell lines and cell culture

The hICD construct containing the intracellular domain of human FPC (192 aa) was described previously [9]. The hTMC construct containing the human FPC C-terminal 225 aa was made by RT-PCR and cloned into pcDNA4 at *Hind III/Xba I* sites (Figure S1). For the establishment of stable cell lines, hICD construct or empty vector was transfected with FuGENE6 (Roche, Madison, WI, USA) into mIMCD-3 cells (ATCC, Bethesda, MD, USA) cultured in DMEM/F12 media (Cellgro, Manassas, VA, USA), supplemented with 10% FBS (Invitrogen), and screened for cells resistant to Zeocin (Invitrogen, 750 µg/ml). For amino acid deprivation condition, cells were serum-starved for 16 h and then cultured in DMEM/F12 media without amino acids for 30 min. For drug treatments, stable cells were incubated with culture media containing appropriate chemical reagents for a period of time as specified in the Results. FPC knockdown cells were described previously [13]. Different amounts of hICD expression construct DNA were transiently transfected into LLC-PK1 cells stably expressing full-length FPC or empty vector control cells P4L [12]. Two days after transfection, cells were harvested for western blotting analyses.

### Immunostaining

The method for immunostaning was described in detail previously [13]. In brief, cultured cells were fixed with 3% paraformaldehyde (Sigma)/2% sucrose (Sigma) and permeabilized with 0.1% Triton X-100 (Sigma). After incubation with primary antibodies for 1 h, cells were incubated with secondary antibodies Alexa Fluor 488 and/or 594 (Invitrogen) for another hour. Nuclei were stained with DAPI (4′-6-diamidino-2-phenylindole) (Sigma). A Zeiss Axioskop2 Plus fluorescence microscope (Carl Zeiss Inc., Thornwood, NJ, USA) and the Spot camera system (Diagnostic Instruments, Sterling Heights, MI, USA) were used for epifluorescence analysis.

### Protein extraction, co-immunoprecipitation, and immunoblotting

Proteins were extracted with M-PER Mammalian Extraction Reagent (Pierce, Rockford, IL, USA) or RIPA lysis buffer (Upstate, Waltham, MA, USA) according to the protocol provided by the company. For immunoprecipitation, the supernatants were precleared with protein A/G agarose beads (Pierce; Invitrogen) for 1 h at +4°C before addition of the primary antibody. Subsequently, agarose beads and the antibody were added and incubated at +4°C for 4–24 h, followed by centrifugation at 10,000 g for 1 min at +4°C. The beads were washed twice with the lysis buffer. Finally, 50 µl of Laemmli's SDS-Sample buffer (Boston BioProducts, Ashland, MA, USA) was added. The protein samples were electrophoresed in a 5%–12% acrylamide Laemmli resolving gel and transferred to Hybond ECL nitrocellulose membranes (GE Healthcare, Boston, MA, USA). After blocking with 5% non-fat dry milk (Bio-Rad, Hercules, CA, USA) in PBS, the filter was incubated with the primary antibody and washed with PBS/0.1% Tween 20 (Bio-Rad). The filters were finally incubated with secondary immunoglobulins conjugated with HRP (GE Healthcare). The bound antibodies were detected with the Detection Reagent (Pierce). Restore Western Blot Stripping Buffer (Pierce) was used according to the protocol provided by Pierce to strip the membrane, prior to reblotting with another antibody. Quantification of the band intensity was accomplished with NIH Image J software. Human kidney samples were gifts from Dr. P. Wilson. The ages of three ARPKD patients were 2 years, 1 month and 9 days, and three normal controls were 2 years, 15 months and 2 months, respectively. These samples had been published previously [32].

### Cell growth curve and flow cytometry

Cells were trypsinized, pelleted, and resuspended in 5 ml of culture medium, followed by seeding in 10 cm dishes with a density of ∼50,000 cells per dish. Cells were stained with tryptophan blue (Invitrogen) and counted with a hemocytometer daily for a total of 9 days. Cells in the culture media were also counted and included. Cell cycle analysis was performed with flow cytometry (BD FACSCanto™ benchtop flow cytometer, Franklin Lakes, NJ, USA). For cell synchronization, cells were cultured in medium without FBS for 48 h, followed by addition of 10% FBS for 24 h. Before flow cytometry, cells were stained with propidium iodide (Invitrogen)/Triton X-100 staining solution with RNase A (Invitrogen) for 30 min at room temperature.

### Apoptosis assay

Apoptosis was evaluated with flow cytometry and TUNEL staining. TUNEL staining was done according to the protocol provided by Roche (In Situ Cell Death Detection Kit). Briefly, cells cultured on the plates were fixed with 3% paraformaldehyde with 2% sucrose for 10 min, followed by complete washing. Before TUNEL reaction mixture was added, cells were permeabilized with Triton X-100. Samples were analyzed by fluorescence microscopy as described above.

### Tubulogenesis assay in 3D collagen gels and confocal microscopy [33], [34]

Tubulogenesis was performed in PureCol gels (Sigma). Cells were trypsinized and resuspended in 1 ml of DMEM/F12 medium. Five to ten microliter of single suspension (∼4×10^4^ cells) was well mixed with 850 µl of collagen solution (collagen type I, 10× DMEM (Sigma), HEPES (Sigma) at 8∶1∶1 ratio). Hepatocyte growth factor (Sigma) was added into the solution before seeding (0.5 µg/ml) or into the culture medium in some experiments. Collagen solution was poured into a 6-well plate for 1–2 h for solidification followed by addition of DMEM/F12/10% FBS medium. Cells were allowed to grow in a collagen gel for up to 8 days before staining. For confocal microscopy analysis, the gel was first incubated with 1× collagenase (Sigma) for 10 min at 37°C before fixation with 3% PFA for 30 min, followed by addition of the quench solution (0.7 M NH_4_Cl and glycine in PBS) for 10 min. After washing, acetylated α-tubulin antibody (1∶10,000) in PFS (14% fish skin gelatin and 0.2% saponin in PBS) was incubated with the gel overnight at +4°C. FITC-labeled secondary antibody and rhodamine phalloidin (1∶250) (Invitrogen) were incubated with the gel for 1 h at room temperature. Before mounting, the gel was incubated again with 3% PFA for 15–20 min, followed by washing and DAPI staining procedures. Confocal images were captured with Nikon microscopy (eclipse 80i) and analyzed with software EZ-C1 (3.91).

### Statistics

Values were expressed as mean ± standard deviation (SD) or standard error (SE). Student's T-Test and Chi-Square Test were used. Spearman correlation was used to calculate the correlation coefficient of time and hICD expression level. p<0.05 was considered statistically significant.

## Supporting Information

Figure S1 **Schematic structure of the human FPC C-tail construct used in this study.** Top, scheme of human full-length FPC which encodes a 4,074-aa protein with a large extracellular domain, a single transmembrane domain (red box), and a 192-aa intracellular fragment; Middle, human FPC C-tail hICD construct which contains the entire 192-aa intracellular domain and a 9-aa myc-tag (red oval). Bottom, mouse C-tail [Ref. 16] is shown for comparison. Green letters, partial or entire ciliary localization signal; Blue box, nuclear localization signal.
(TIF)
