# Supplementary figures and images for: The Cytoplasmic Tail of FPC Antagonizes the Full-Length Protein in the Regulation of mTOR Pathway
Source: PLoS One. 2014 May 22;9(5):e95630. doi: 10.1371/journal.pone.0095630 (PMC4031230; doi:10.1371/journal.pone.0095630)

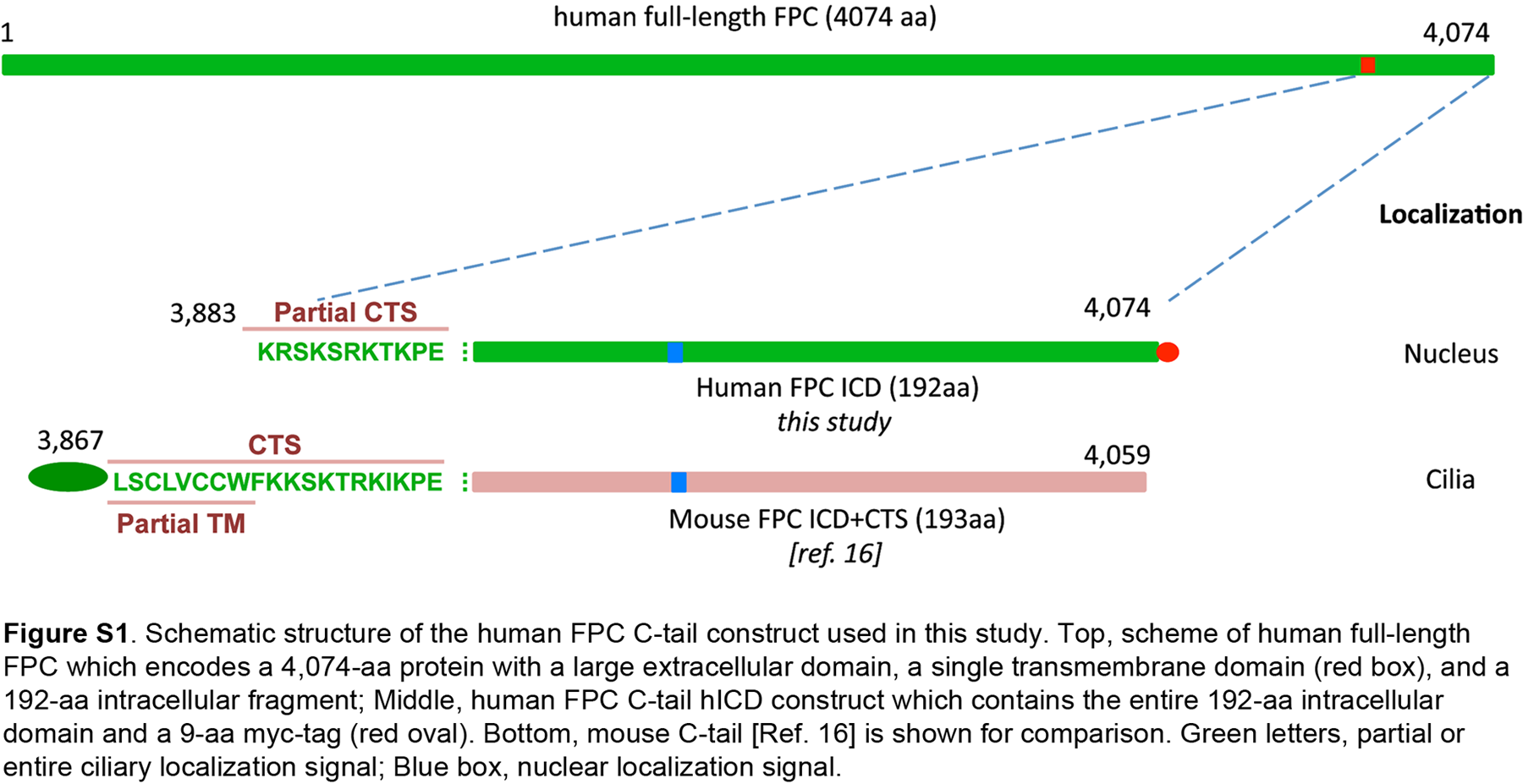

Supplement: Figure S1 — Schematic structure of the human FPC C-tail construct used in this study. Top, scheme of human full-length FPC which encodes a 4,074-aa protein with a large extracellular domain, a single transmembrane domain (red box), and a 192-aa intracellular fragment; Middle, human FPC C-tail hICD construct which contains the entire 192-aa intracellular domain and a 9-aa myc-tag (red oval). Bottom, mouse C-tail [Ref. 16] is shown for comparison. Green letters, partial or entire ciliary localization signal; Blue box, nuclear localization signal. (TIF) [file pone.0095630.s001.tif]
